# Global phenotypic and genomic comparison of two *Saccharomyces cerevisiae* wine strains reveals a novel role of the sulfur assimilation pathway in adaptation at low temperature fermentations

**DOI:** 10.1186/1471-2164-15-1059

**Published:** 2014-12-03

**Authors:** Estéfani García-Ríos, María López-Malo, José Manuel Guillamón

**Affiliations:** Departamento de Biotecnología de los alimentos, Instituto de Agroquímica y Tecnología de los Alimentos (CSIC), Avda. Agustín Escardino, Po Box 73E-46100, Paterna Valencia, Spain; Biotecnologia Enològica. Departament de Bioquímica i Biotecnologia, Facultat de Enologia, Universitat Rovira i Virgili, Marcel•li Domingo s/n, 43007 Tarragona, Spain

**Keywords:** Wine yeast, Cold adaptation, Transcriptomics, Proteomics, Genomics, Oxidative stress, Glutathione biosynthesis, Genotype-phenotype association

## Abstract

**Background:**

The wine industry needs better-adapted yeasts to grow at low temperature because it is interested in fermenting at low temperature to improve wine aroma. Elucidating the response to cold in *Saccharomyces cerevisiae* is of paramount importance for the selection or genetic improvement of wine strains.

**Results:**

We followed a global approach by comparing transcriptomic, proteomic and genomic changes in two commercial wine strains, which showed clear differences in their growth and fermentation capacity at low temperature. These strains were selected according to the maximum growth rate in a synthetic grape must during miniaturized batch cultures at different temperatures. The fitness differences of the selected strains were corroborated by directly competing during fermentations at optimum and low temperatures. The up-regulation of the genes of the sulfur assimilation pathway and glutathione biosynthesis suggested a crucial role in better performance at low temperature. The presence of some metabolites of these pathways, such as S-Adenosilmethionine (SAM) and glutathione, counteracted the differences in growth rate at low temperature in both strains. Generally, the proteomic and genomic changes observed in both strains also supported the importance of these metabolic pathways in adaptation at low temperature.

**Conclusions:**

This work reveals a novel role of the sulfur assimilation pathway in adaptation at low temperature. We propose that a greater activation of this metabolic route enhances the synthesis of key metabolites, such as glutathione, whose protective effects can contribute to improve the fermentation process.

**Electronic supplementary material:**

The online version of this article (doi:10.1186/1471-2164-15-1059) contains supplementary material, which is available to authorized users.

## Background

In natural environments with diurnal and/or seasonal temperature changes, temperature is one of the main relevant environmental variables that microorganisms, including yeast species, have to deal with. Temperature is also a key factor in some industrial processes involving microorganisms. Low temperatures (10-15°C) are used in wine fermentations to enhance production and to retain flavor volatiles. In this way, white and “rosé” wines of greater aromatic complexity can be achieved [1, 2]. Yeast undergoes considerable stress during wine fermentation due to the high concentrations of sugars in grape must, which leads to high osmotic pressure at the beginning of the process. Then as fermentation proceeds, ethanol accumulation, limiting nitrogen concentration, or even presence of SO_2_, imposes further pressure on wine yeast. Therefore to these difficulties, which are inherent to the process, we should add a sub-optimal temperature for the primary fermentation agent. Temperatures below its optimum range for growth, around 32°C [3], affect both the yeast growth and fermentation rates, and give rise to not only a prolonged lag phase, but also to the production of stuck and sluggish fermentations [4]. Low temperature has several effects on biochemical and physiological properties in yeast cells: poorly efficient protein translation; low fluidity membrane; changes in lipid composition; slow protein folding; stabilization of mRNA secondary structures; reduced enzymatic activities [5–8]. These problems can be avoided by providing better-adapted yeasts to ferment at low temperature. In past years, some attempts have been made to elucidate the response to cold in *Saccharomyces cerevisiae* through a variety of high-throughput methodologies. Some studies have analyzed the genome-wide transcriptional response of *S. cerevisiae* to low temperatures. These studies have focused mainly on cold shock [6, 7, 9, 10]. Schade et al. [7] identified two distinct phases in the cold shock response: 1) an early cold response (ECR) occurring within the first 12 h after exposure to low temperature; 2) a late cold response (LCR) taking place beyond 12 h after exposure to low temperature. An ECR induces the genes implicated in RNA and lipid metabolism, whereas the genes induced during an LCR encode mainly the proteins involved in protecting the cell against a variety of stresses. In fact, the LCR response is very similar to the general stress response mediated by the transcription factors Msn2p/Msn4p. However, the response type depends on the duration of exposure to stressful conditions. Sudden exposure to environmental changes (e.g., cold shock) is likely to trigger a rapid, highly dynamic stress response (adaptation). Prolonged exposure to nonlethal stimuli leads to acclimation; i.e., establishment of a physiological state in which regulatory mechanisms, like gene expression, fully adapt to suboptimal environmental conditions [8]. Tai et al. [8] compared their transcriptomic results obtained during cold acclimation in a steady-state chemostat culture with other previous genome-wide transcriptional studies of batch cultures at low temperature, and found major discrepancies among low-temperature transcriptome datasets. These authors partially explained these major differences by the cultivation method used in different transcriptome experiments. Although batch cultures are well-suited to study low temperature adaptation dynamics, they are poorly adapted to study prolonged exposure to low temperature. In such cultures, the specific growth rate (μ) is strongly affected by temperature, which makes it impossible to dissect temperature effects on transcription from specific growth rate effects. Two recent chemostat studies [11, 12] also found that the growth rate itself has a strong effect on transcriptional activity. Furthermore, chemostat cultures help to accurately control the specific growth rate, so the concentration of all the metabolites is constant over time, thus providing a good platform to study microbial physiology, proteome profiles and gene expression [8].

Other recent studies of our group analyzed the changes in the proteomic profile [13, 14] and in the metabolome [15] due to low temperature. Nine proteins were identified as representing the most significant changes in proteomic maps during the first 24 h of fermentation at low (13°C) and standard (25°C)temperatures. These proteins were involved mainly in oxidative stress response and glucose and nitrogen metabolism. In the global metabolic comparison, the main differences in the *S. cerevisiae* strain growing at low temperature were metabolites related with lipid metabolism and redox homeostasis.

So far, none of these previous studies have tackled adaptation at low temperature using a global approach, which involves differences at the genomic, transcriptomic and proteomic levels of two commercial wine strains, selected on the basis of a significant divergent phenotype growing at low temperature. In the first stage of the work, these strains were selected from a collection of 27 commercial *S. cerevisiae,* which were grown at temperatures ranging from 4 to 45°C in both minimal media (SD) and synthetic must (SM). The fitness differences at low temperature of these selected strains were confirmed in a competition experiment during wine fermentation. In a second stage, the aim was to decipher the molecular basis underlying this divergent phenotype by analyzing the genomic, proteomic and transcriptomic differences between both strains at low temperature. The up-regulation of the genes of the sulfur assimilation pathway and glutathione biosynthesis suggested a crucial role in better performance at low temperature. The presence of some metabolites of these pathways, such as S-adenosilmethionine (SAM) and glutathione, counteracted the differences in growth rate at low temperature in both strains. Generally, the proteomic and genomic changes observed in both strains also supported the importance of these metabolic pathways in adaptation at low temperature.

## Results

### Effect of temperature on wine yeast growth and selection of two strains with different growth behavior at low temperature

**Figure 1:**
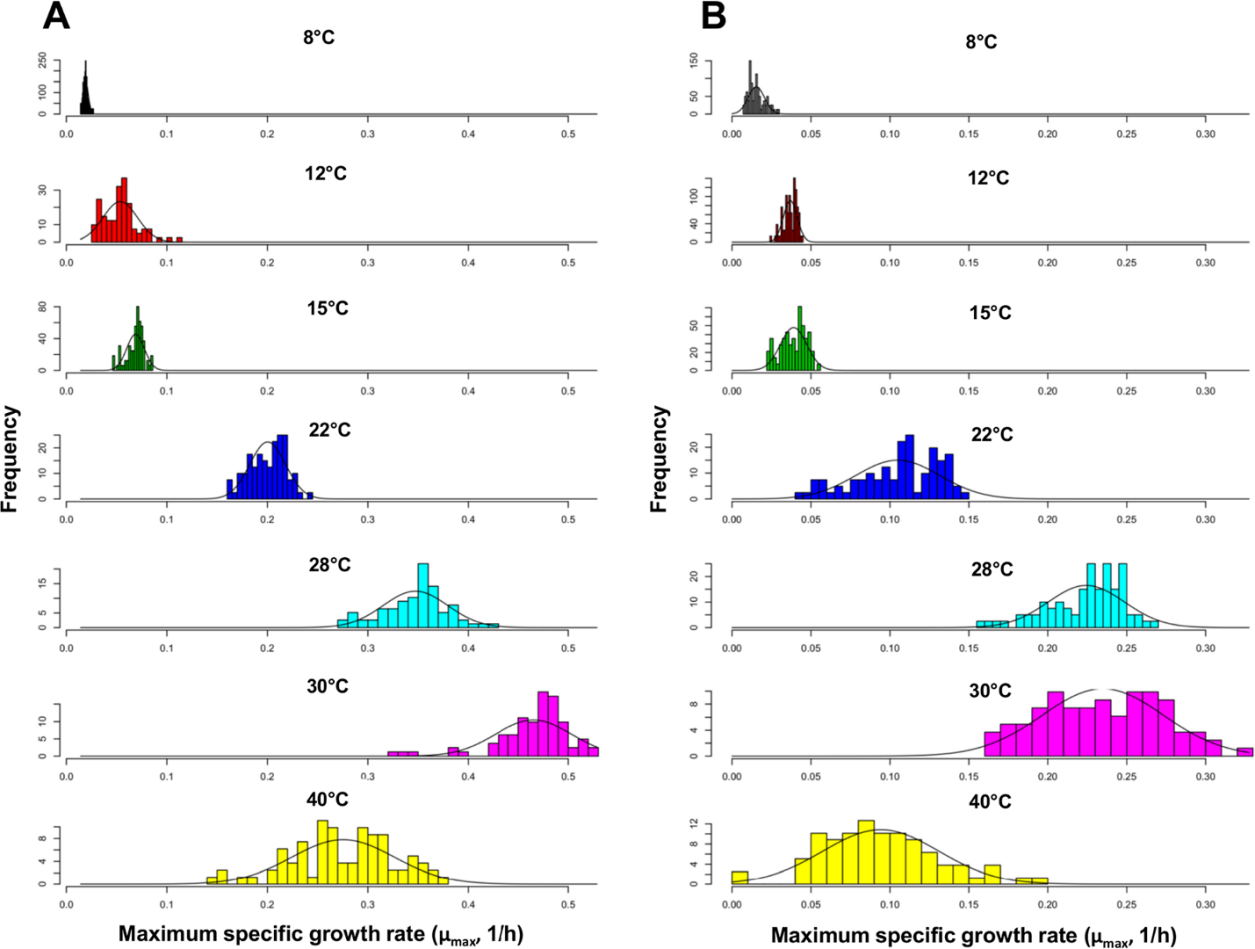
**Histogram of the distribution of the maximum specific growth rate μ**_**max**_ **(h** ^**-1**^ **) according to the temperature in two media: SD (A) and SM (B).** Bars represent the frequency of the individuals with an μ_max_ value within the same range of variation. The superimposed bell-shaped line shows the growth data at different temperatures following a Gaussian distribution. Due to differences in the growth rate, different scales were used for μ_max_ distribution in SD and SM.

In order to select two strains with different growth behavior at low temperature, we tested growth capacity at different temperatures with a collection of commercial strains (Additional file 1: Table S1). Figure 1 shows the global distribution of μ_max_ for all 27 strains at different temperatures in SD and SM. This representation follows a normal (or Gaussian) distribution in which the μ_max_ values were lower and variance was wider in SM (Figure 1B) than in SD (Figure 1A). In addition, the higher temperature was, the wider variance became. For the temperatures assayed, the average optimum temperature of these strains can be settled at around 33°C for SD and somewhat lower for SM.

Similar conclusions were drawn from the boxplot representation of each individual strain if compared to the average μ_max_ of the 27 strains within the whole temperature range assayed (Additional file 2: Figure S1). Dispersion in these boxplots was much greater for each strain growing in SM than in SD. This representation also revealed the general fitness of each strain in comparison to the average fitness of all the strains and temperatures.

**Figure 2:**
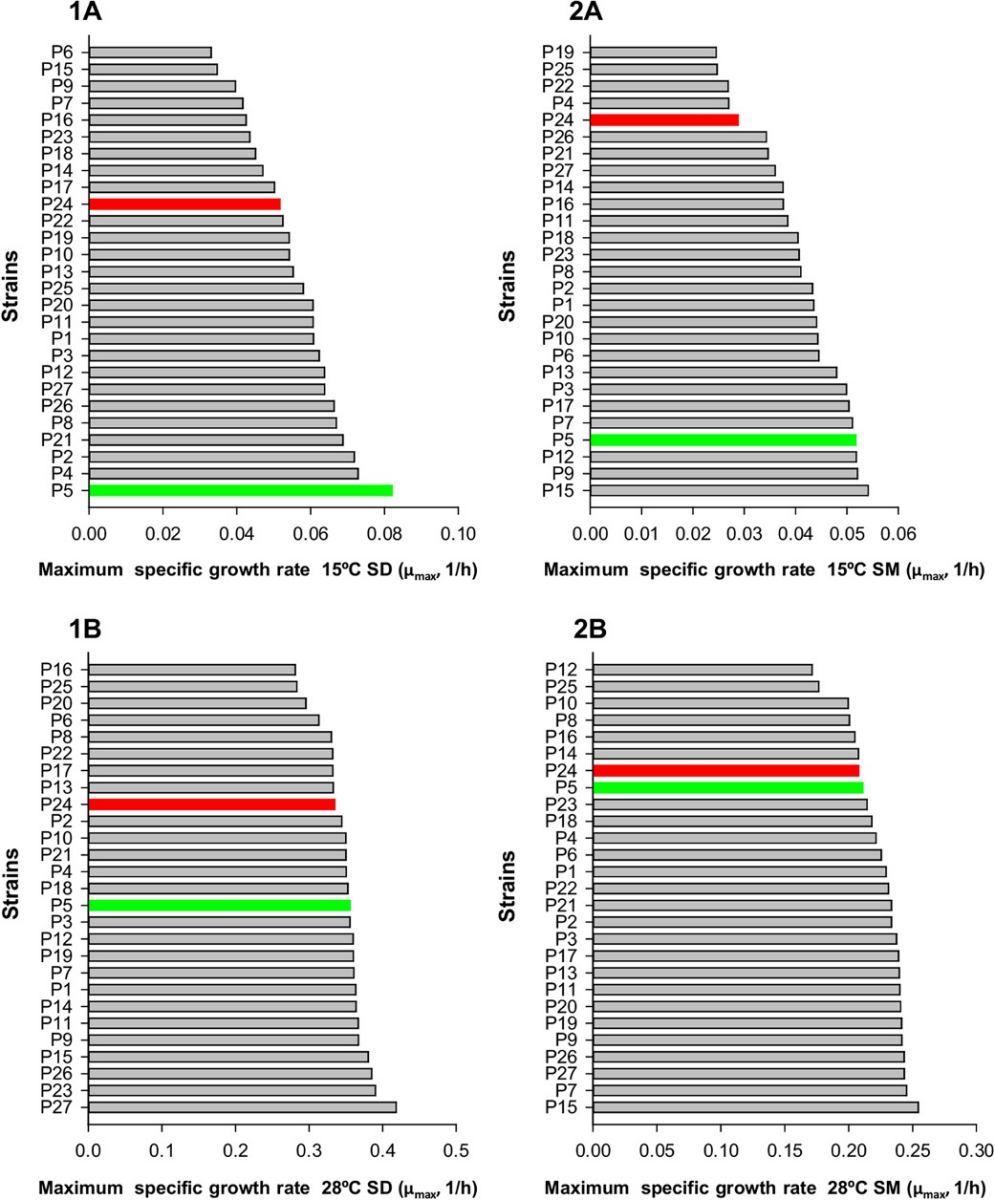
**Selection of strains with a divergent phenotype at low temperature.** Strains P5 (green) and P24 (red) were selected on the basis of the μ_max_ (h^-1^) in SD **(A)** and SM **(B)** at 15°C **(1)** and 28°C **(2)**. Due to differences in the growth rate, different scales were used for μ_max_ distribution in SD and SM.

Considering the whole data set at different temperatures, a decision was made to take 28°C as the optimum reference temperature and 15°C as the reference temperature for cold. These temperatures showed the biggest differences between strains and media. The μ_max_ values were used to select two strains with evident different growth behavior at low temperature, regardless of the growth medium, but with no significant differences at the optimum temperature. Following these selection criteria, P5 and P24 were chosen as the candidate strains with good and bad growth behavior at low temperature, respectively (Figure 2). P5 showed the best growth performance at low temperature in SD and one of the best ranked in SM. Conversely, P24 was ranked among the strains with a lower μ_max_ value in both media. P5 corresponds to commercial strain Lalvin®ICVGRE, which is recommended for temperature fermentations ranging from 15 to 30°C by the marketer.

### Fermentation kinetics and competition analysis of the selected strains

In order to evaluate whether the higher μ_max_ values growing in a miniaturized system correlated well with higher fitness during alcoholic fermentation, a competition experiment was performed under microvinification conditions between the “good” and “bad” strains at the low and optimum temperatures. In order to make monitoring competitive capacity during fermentation easier, a P5 reporter strain was constructed by deleting one copy of the open reading frame (ORF) of gene *GAL1* and replacing it with the deletion cassette GFP-*KanMX4.* This system was based on the expression of the green fluorescent protein (GFP) under the control of the *GAL1* promoter, a gene which is absolutely repressed during wine fermentation and activated in the presence of galactose. The cells from the fermentation culture were incubated directly in YPGal to determine the percentage of fluorescent cells by flow cytometry. The SM fermentations were inoculated with either a pure culture of each strain or a mixture of both strains. In order to verify that the deletion of one copy of the *GAL1* gene did not affect the fitness of strain P5, the fermentations inoculated with either the reporter P5-GFP strain or a mixture of the parental P5 and the reporter P5-GFP strains were also carried out. The kinetics of these fermentations was estimated by calculating the time needed to ferment 5% (T5), 50% (T50) and 100% (T100) of sugars in SM (Table 1). T5, T50 and T100 approximately matched the beginning (lag phase), middle (end of the exponential phase) and end of fermentation, respectively. As expected, the replacement of *GAL1* gene with a GFP cassette did not modify the fermentation fitness of strain P5. Surprisingly, P5-GFP finished its fermentation a few hours earlier than its wild-type P5 (Table 1). Moreover, the percentage of each strain was kept at around 50% throughout the mixed fermentation (P5 GFP/P5) (data not shown).

**Table 1:** **Time (hours) required to consume the sugar content in a synthetic grape must**

Yeast strain	Temperature	T5	T50	T100
	**15°C**	23.06 ± 3.84	221.65 ± 8.00	889.18 ± 19.34
**P5**	**28°C**	12.93 ± 0.28	44.53 ± 0.32	118.87 ± 2.59
	**15°C**	30.75 ± 0.00^a^	395.90 ± 58.03^a^	930.15 ± 0.00^a^
**P24**	**28°C**	21.28 ± 2.61^a^	47.53 ± 1.97	118.66 ± 3.53
	**15°C**	25.18 ± 1.10	188.98 ± 9.67^a^	869.96 ± 2.21^a^
**P5GFP**	**28°C**	13.78 ± 2.93	47.53 ± 0.56	87.93 ± 1.62
	**15°C**	28.18 ± 4.43	210.51 ± 2.93	869.96 ± 2.21^a^
**P5 GFP/P5**	**28°C**	14.90 ± 0.97^a^	46.21 ± 2.27	121.12 ± 6.32
	**15°C**	50.67 ± 0.00^a^	270.98 ± 6.23^a^	1125.79 ± 0.00^a^
**P5 GFP/P24**	**28°C**	21.84 ± 0.58^a^	53.71 ± 4.60^a^	126.37 ± 2.12^a^

These values are the mean ± SD of three independent experiments.

T100 = time to reach a density of ≤ 998 g/L.

^a^Significant differences in relation to the control strain (P5).

**Figure 3:**
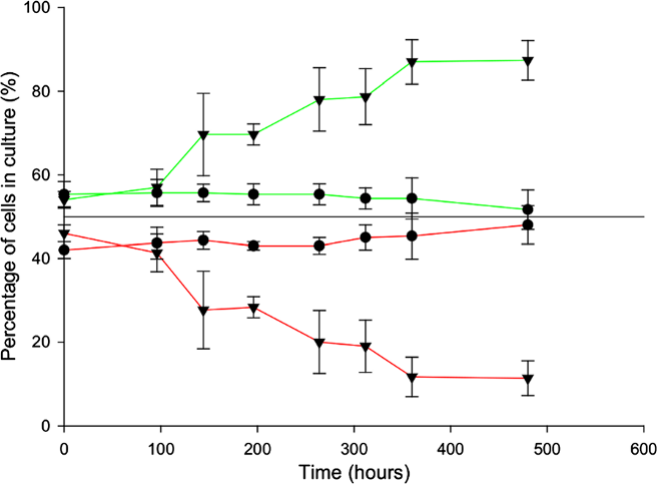
**Population dynamics of a mixed culture between strains P5 (green lines) and P24 (red lines) growing in synthetic must (SM).** The percentage of each strain was determined by flow cytometry during fermentation (0, 24, 48, 72, 96,144, 240 and 480 h) at 15°C (▲) and 28°C (●).

Strain P24 underwent significant delays in all the low-temperature fermentation stages if compared to the P5 fermentations, whereas only the beginning of fermentation (T5) was delayed at 28°C. It is noteworthy that the interaction of both strains in the mixed fermentation (P5 GPF/P24) significantly affected fermentation lengths at both temperatures, with long delays noted in the fermentation ends if compared to P5 fermenting as a pure culture. This result was even more surprising when the percentage of each strain was monitored during these mixed fermentations (Figure 3). Strain P5 gradually took over the fermentation process at low temperature and obtained percentages of around 85% of the total population at the end of the process. Conversely no strain dominated the fermentation process at 28°C, with populations of around 50% for each strain. The greater competition capacity of strain P5 at low temperature was also corroborated by repeating this experiment in SD medium (Additional file 3: Figure S2).

### Transcriptomic analysis revealed a key role of the sulfur assimilation pathway in adaptation at low temperature

**Figure 4:**
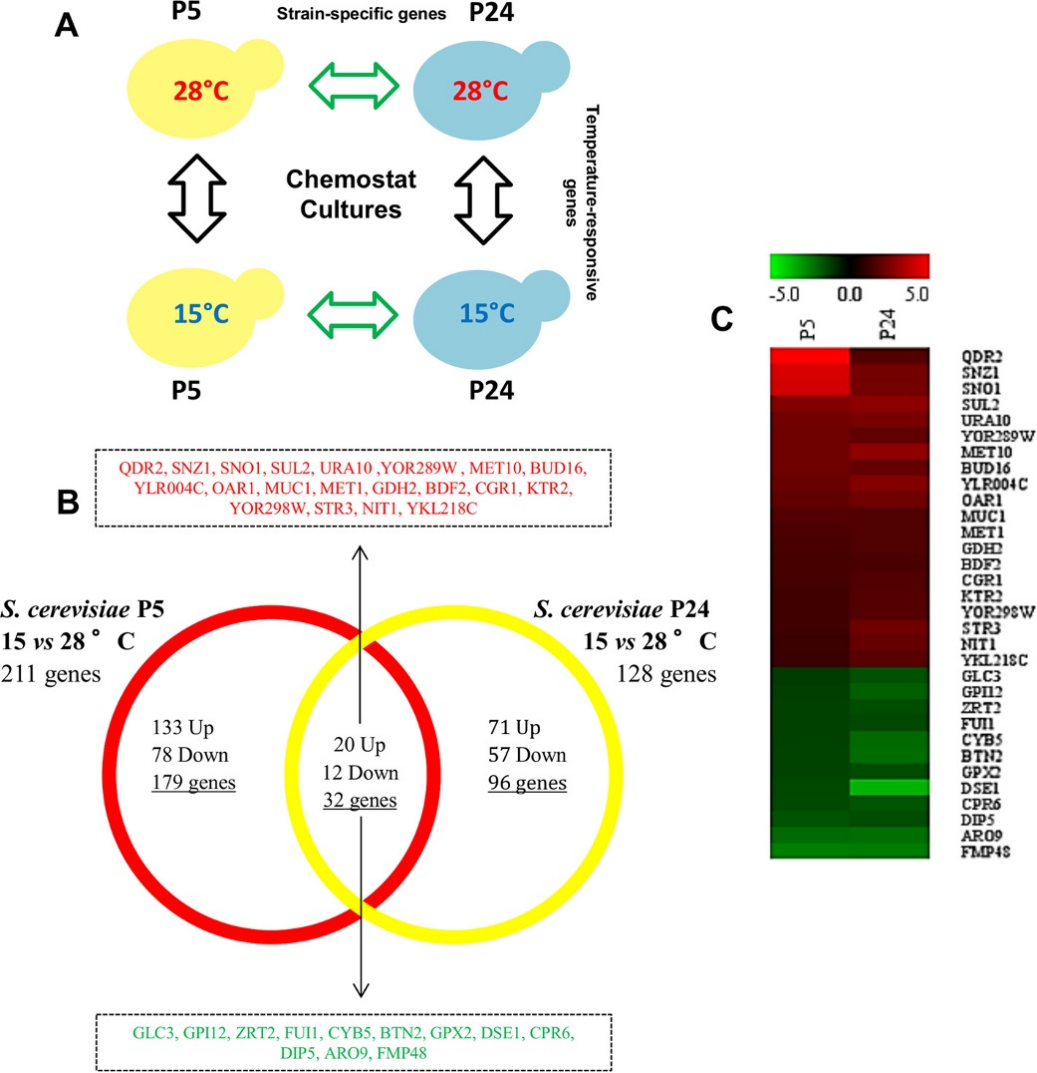
**The transcriptional response at low temperature in the two selected strains. (A)** Scheme of the experimental design: the transcriptomic changes in the same strain are due to growth temperature (temperature-responsive genes) or the transcriptomic changes at the same temperature depend on the wine strain (strain-specific genes). **(B)** Venn’s diagram of the temperature-responsive genes in both strains. The common genes among strains are highlighted. Red indicates up-regulated genes, while green denotes down-regulation. **(C)** Heat map depicting the level of expression of the common genes in both strains at low temperature.

To compare the transcriptome of P5 and P24 grown at 15°C and 28°C with no interference by the different maximum specific growth rate at these two temperatures, these strains were grown in chemostat cultures at a fixed dilution rate of 0.028 h^-1^ at both temperatures. This dilution rate corresponded to the μ_max_ of the strain P24 growing at 15°C. This experimental design allowed us to compare the transcriptional differences due to temperature in each strain (temperature effect) and the differences between strains at the same temperature (strain effect) (Figure 4A). A list of genes differentially expressed when comparing temperatures and strains and the MIPS functional categories analysis of these genes is provided as supplementary files (Additional file 4: Table S2, Additional file 5: Table S3, Additional file 6: Table S4, Additional file 7: Table S5, Additional file 8: Table S6).

The comparison made by temperatures revealed that strains P5 and P24 had 211 and 128 differentially regulated genes at low temperature, respectively (Figure 4B). This temperature response was mainly strain-dependent because only 32 genes were commonly regulated in both strains (Figure 4B). Although these common genes showed a consistent up- or down-regulation in both strains, differences in the expression level were also observed in a simple view of the heat-map (Figure 4C). As these genes should play a crucial role in adaptation at low temperature, clear differences in their activity can explain the different phenotypic behavior of both strains at low temperature. For instance, genes *QDR2, SNZ1*and *SNO1* were much more markedly up-regulated by low temperature in P5 than in P24. *QDR2* is a plasma membrane transporter involved in the K^+^ homeostasis induced by nitrogen limitation [16], whereas *SNZ1* and *SNO1* are involved in pyridoxine metabolism, which is essential for sphingolipids biosynthesis, and are also up-regulated in response to nutrient starvation [17]. Conversely, genes *GPI12*, *CYB5, BTN2* and *DSE1* were more strongly down-regulated in P24 than in P5. *GPI12* is involved in the synthesis of glycosylphosphatidylinositol (GPI), the most important anchor of plasma membrane proteins [18]. *CYB5* is involved in the sterol and lipid biosynthesis pathways, and acts as an electron donor to support sterol C5-6 desaturation [19]. *BTN2* modulates arginine uptake [20] and *DSE1* is involved in cell wall organization.

**Figure 5:**
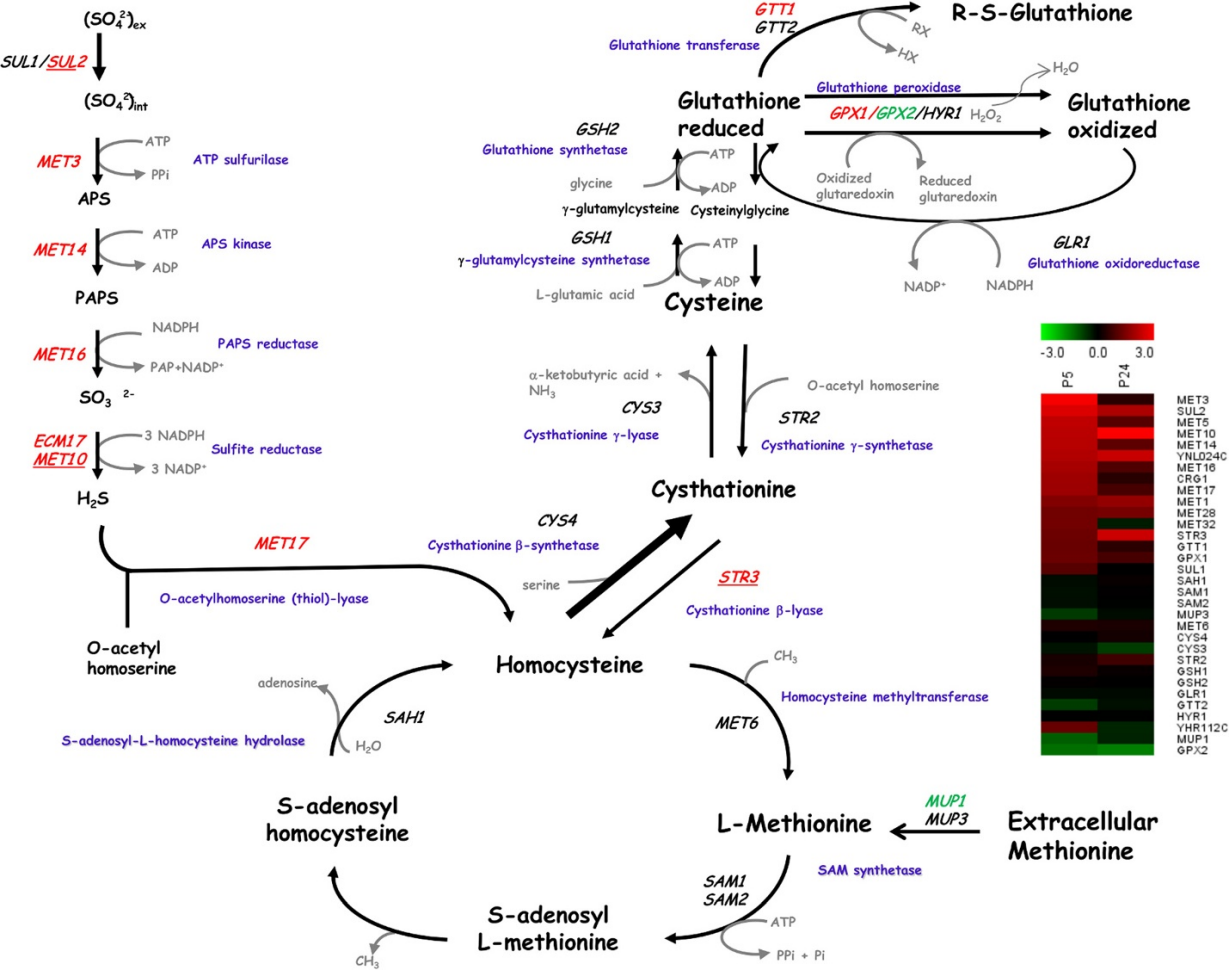
**The sulfur assimilation pathway and the genes encoding the enzymes of the different biosynthetic steps.** The genes in red and green represent up-regulation and down-regulation in strain P5 at low temperature, respectively. Underlined genes mean identical regulation in P24. Heat map shows the expression of the genes of this pathway that were differentially expressed in both strains.

One interesting common trait in all the comparisons made of the transcriptional changes observed either by temperature or strains was the presence of functional categories “nitrogen, sulfur and selenium metabolism”, “sulfur metabolism” and “metabolism of methionine” in the genes up-regulated at low temperature (Table 2). The genes included in these functional categories belong mainly to the sulfur assimilation pathway (Figure 5). This pathway incorporates extracellular sulfate into several key sulfur-containing compounds; i.e.; homocysteine, methionine, S-adenosylmethionine or glutathione [21, 22]. However, clear differences were observed in the transcriptomic activation of this route in both strains. Whereas most genes were overexpressed in strain P5, very few were also up-regulated in P24.

**Table 2:** **Functional group analysis of common up-regulated MIPS categories of the transcriptomic data comparison**

Sample	No. of genes	MIPS			
		Name	No. of genes	***p***-value	Example of genes
		Metabolism of methionine	10	2 · 10^-6^	ALT1:DAL7:DUR80
					GDH2:MET1:MET3
**P5 15°C-28°C**	32	Nitrogen, sulfur and selenium metabolism	16	1 · 10^-6^	MET5:MET10:MET14
		Sulfur metabolism	6	1 · 10^-5^	MET16:MET17:MET28
					MET32:NIT1:STR3:YHR112C
		Metabolism of methionine	3	7 · 10^-3^	ATO3:GDH2:MET1
**P24 15°C-28°C**	11	Nitrogen, sulfur and selenium metabolism	6	1 · 10^-3^	MET10:NIT1:STR3
		Sulfur metabolism	2	3 · 10^-3^	
		Metabolism of methionine	3	1.99 · 10^-2^	ASP3:DAL7:FMO1
**P5-P24 15°C**	12	Nitrogen, sulfur and selenium metabolism	7	1 · 10^-4^	MET3:MET14:MET32
		Sulfur metabolism	2	7.1 · 10^-3^	OPT1
		Metabolism of methionine	3	1.81 · 10^-4^	GDH2:MET1:MET10
**Common key genes 15°C**	10	Nitrogen, sulfur and selenium metabolism	5	1.14 · 10^-5^	NIT1:STR3
		Sulfur metabolism	2	2.79 · 10^-4^

### The concentration of glycolytic and stress oxidative proteins increased at low temperature

A proteome analysis of the same samples used for the transcriptomic analysis was done to evaluate changes in proteins as a result of low temperature in each strain. As done previously, comparisons of 2D-PAGE gels were made to detect temperature-dependent and strain-dependent protein changes. Around 200 spots were detected on 2-D gels in both strains growing at 28°C (Additional file 9: Table S7). This number of detected spots increased to 236 and 251 for strains P5 and P24, respectively, when cells were grown at low temperature. More interestingly, most of these detected proteins were matched on both 2-D gels but, between matched spots, very few showed statistically significant increases or decreases (Additional file 9: Table S7 and Table 3).

**Table 3:** **Proteins whose concentration increased (positive numbers) or decreased (negative numbers) by at least 2-fold at 15°C**

Strain	Gene name	Protein name	Metabolic function	Fold change
**P5**	*TPI1*	Triosephosphateisomerase	Glycolisis	27.28
	*AHP1*	Peroxiredoxin type-2	Oxidative stress	20.07
	*PDC1*	Pyruvate DeCarboxylase	Glycolisis, Glucose fermentation	13.95
	*TSA1*	Thioredoxin peroxidase	Oxidative stress	5.28
	*CDC19*	Pyruvatekinase	Glycolisis, Glucose fermentation	5.16
	*ENO2*	Enolase II, phosphopyruvatehydratase	Glycolisis	3.28
	*FBA1*	Fructose 1,6-bisphosphate aldolase	Glycolisis	2.15
**P24**	*RPL31A*	Ribosomal Protein of the Large subunit	Structural constituent of ribosome	17.65
	*TDH1*	Glyceraldehyde-3-phosphate dehydrogenase	Glycolisis	7.11
	*TEF1*	Translational elongation factor EF-1 alpha	Translation	5.12
	*TDH3*	Glyceraldehyde-3-phosphate dehydrogenase	Glycolisis	4.76
	*GPM1*	Glycerate phosphomutase	Glycolisis	3.53
	*ENO2*	Enolase II, phosphopyruvatehydratase	Glycolisis	-4.09

The concentration of seven proteins increased at low temperature in P5 (Table 3); five were involved in glycolysis and glucose fermentation (Fba1p, Tpi1p, Eno2p, Cdc19p and the key fermentative enzyme Pcd1p), mainly belonging to the lower part of the glycolysis, the other two proteins were involved in oxidative stress (Ahp1p, Tsa1p). Tsa1p is a physiologically important antioxidant protein that is useful as enzymic defense against sulfur-containing radicals [23], thus providing protection against an oxidation system without thiol. Ahp1p is a similar peroxiredoxin to Tsa1p that forms a disulfide-linked homodimer upon oxidation, and *in vivo* requires the presence of a thioredoxin system to perform its antioxidant protective function. Unlike Tsa1p, which is specific for H_2_O_2_, Ahp1p is also specific for organic peroxides [24]. This latter protein shows one of the largest increases (20-fold or more) at low temperature. Regarding strain P24, three of the proteins with increased levels were implicated in the lower part of glycolysis and glucose fermentation (Tdh3p, Tdh1p and Gpm1p). Other proteins with increased levels at low temperature were Tef1p, a translational elongation factor and Rpl31Ap, a ribosomal 60S subunit protein, and both are implicated in translation. Only Eno2p showed significant changes in both strains, but in opposite directions. Low temperature increased the concentrations in P5, but lowered it in P24.

When examining the protein changes in strain P5 if compared to P24 (Table 4), ten proteins showed different concentrations at 15°C, seven of which were more abundant in strain P5. Among these proteins, enzymes of the lower part of glycolysis (Eno2p, Fba1p and Adh1p), proteins implicated in oxidative stress and protein folding (Ahp1p and Ssa2p) and proteins implicated in methionine/cysteine biosynthesis (Met10p and Met17p), were detected. The remaining proteins with lower concentrations in P5 when compared with P24 were Tdh1p, which is induced during heat shock [25], Tdh3p (a key protein to pull the flux through the ATP production stage in the lower part of glycolysis) and Met6p, involved in methionine biosynthesis. At the optimum temperature, only four proteins lowered statistically in P5 (or increased in P24): Ylr179Cp (of unknown function), Gre2p (a reductase implicated in the ergosterol metabolic pathway), Tpi1p (glycolysis) and Sah1p (S-Adenosyl-l-Homocysteine hydrolase involved in methionine biosynthesis).

**Table 4:** **Proteins whose concentration increased (positive numbers) or decreased (negative numbers) by at least 2-fold in P5 in comparison to P24**

Condition	Gene name	Protein name	Metabolic function	Fold change
**15°C**	*MET17*	O-acetylhomoserinesulfhydrilase/	Amino acid biosynthesis (methionine/cysteine)	12.24
	*FBA1*	Fructose 1,6-bisphosphate aldolase	Glycolisis	8.89
	*MET10*	Sulfite Reductase	Sulfate assimilation	6.55
	*ENO2*	Enolase II, phosphopyruvatehydratase	Glycolisis	5.51
	*AHP1*	Peroxiredoxin type-2	Oxidative stress	3.03
	*ADH1*	Alcohol dehydrogenase	Glucose fermentation	2.86
	*SSA2*	Heat shock protein 70	Protein Folding	2.56
	*TDH3*	Glyceraldehyde-3-phosphate dehydrogenase	Glycolisis	-3.11
	*MET6*	Cobalamine-independent Methioninesynthase	Methionine biosynthesis	-3.96
	*TDH1*	Glyceraldehyde-3-phosphate dehydrogenase	Glycolisis	-88.84
**28°C**	*YLR179C*	Unknownfunction	Unknown	-2.08
	*SAH1*	S-Adenosyl-l-Homocysteinehydrolase	Methionine metabolic process	-3.27
	*GRE2*	3-methylbutanal reductase/NADPH-dependent methylglyoxal reductase	Ergosterol metabolic process	-3.50
	*TPI1*	Triosephosphateisomerase	Glycolisis	-9.20

### Addition of SAM and glutathione to SM suppressed growth differences at low temperature

**Figure 6:**
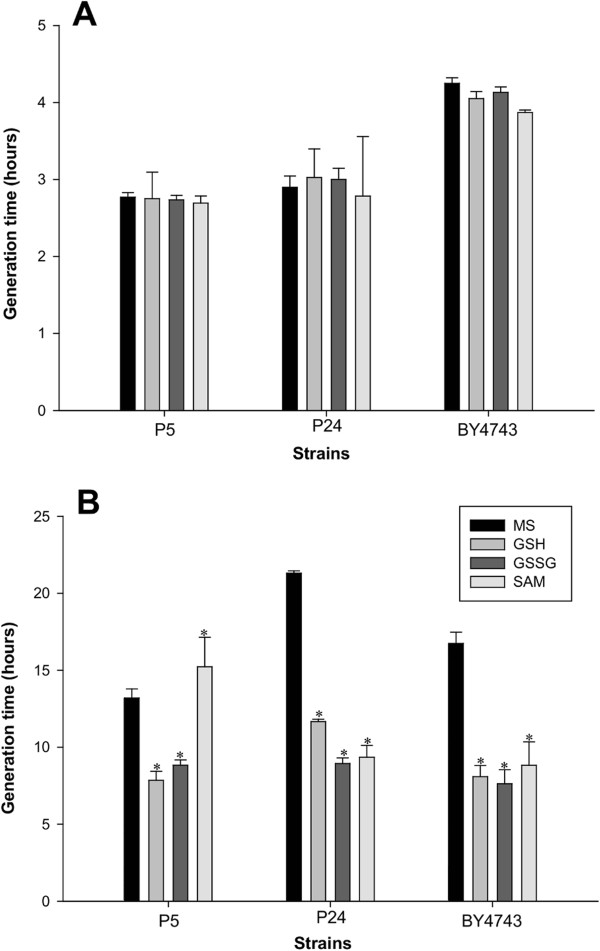
**Generation time (hours) of P5, P24 and lab strain BY4743 at 28°C (A) and 15°C (B).** The assay was carried out in synthetic must (SM) supplemented with different key sulfur-containing compounds (GSH, GSSG, SAM). *Significant differences compared with the strain growing in SM at the same temperature.

As both transcriptomic and proteomic analyses indicated the importance of sulfur metabolism, sulfur amino acid and glutathione biosynthesis, we tested the impact that the addition of the key metabolites of these pathways (SAM, GSH and GSSG) to SM had on wine strain growth (Figure 6). As a control, we also incorporated the lab strain BY4743. Addition of GSH and GSSG significantly shortened the generation time (GT) of P5 at low temperature, while addition of SAM prolonged the GT by 2 h. Quite surprisingly, the presence of the three sulfur-containing compounds dramatically shortened the GT of P24 at low temperature and obtained similar values to these of P5. The behavior of the lab strain BY4743 was similar to P24 as it reduced GT from 17 h to around 8 h. Regarding growth at 28°C, no statistically significant variations were noted in the GT of the three strains when grown in supplemented SM, which indicates a low temperature-dependent effect.

### Strain P5 showed better oxidative stress recovery

The importance of glutathione metabolism and a higher concentration of some peroxiredoxins and thioredoxins at low temperature also suggest the influence of oxidative stress defense on the better fitness of strain P5. To check this implication, both wine strains were incubated in PBS with different H_2_O_2_ concentrations for 1 h at 28°C. After this incubation, the oxidant agent was removed and cells were inoculated in SD and SM. Additional file 10: Figure S3 shows the growth curves at 28°C for each strain after incubation with different H_2_O_2_ concentrations in both media. Increasing H_2_O_2_ concentrations affected mainly the lag phase, which was longer (from 3 to 13 h) the higher the oxidative agent concentration became. The longest lag phases were detected in the P24 growth curves, which revealed worse management or recovery after oxidative shock. Regarding the viability of the strains after incubation at different H_2_O_2_concentrations, and before inoculation in SD or SM, no statistical differences were found between both strains and H_2_O_2_ concentrations (data not shown).

### Whole-genome comparison of the two wine strains

The genomes of the two wine strains were sequenced and compared with that of reference strain S288c (Additional file 11: Figure S4). Based on the raw sequence data, we identified 44532 and 44030 mutations in strain P5 and strain P24, respectively, in comparison to the reference strain. This number of SNPs represents approximately 0.4% of the *S. cerevisiae* genome. When comparing the sequences between both wine strains, the number of SNPs lowered to 6446 mutations, of which 90% gave homozygous changes. According to Liti et al. [26], wine yeasts belong to the same cluster, the Wine/European lineage, while most lab strains, such as S288c, are mosaic strains between the Wine/European cluster and the other four clean lineages. Only 27% of the SNPs between both wine strains represented nonsynonymous changes in the coding region, which resulted in an amino acid change (Additional file 11: Figure S4A). As these nonsynonymous changes could potentially explain the phenotypic differences observed between both strains, we identified mutations in the genes of the sulfur assimilation pathway (Table 5). Many genes of this route presented SNPs which involved amino acid changes in the biosynthetic enzymes and, most importantly, in regulators of the pathway, such as *MET4* and *MET28*. However, conversely to the transcriptomic analysis, the determination of the functional categories with significant overrepresentation among the nonsynonymous SNPs did not include the GO term “sulfur metabolism”, although the related “sulfate/sulfite transport” was included (Additional file 12: Table S8).

**Table 5:** **Genomic changes between strains P5 and P24 in the genes of the sulfur assimilation pathway and glutathione biosynthesis**

Genes	Mutations	Functions
*MET4*	Gln^629^-His	Regulation of sulfur metabolism
*MET28*	Lys^92^-Glu	Regulation of sulfur metabolism
*MET1*	Gly^578^-Asp	Sulfate assimilation and methionine biosynthesis
*GTT1*	Gly^231^-Asp	Glutathione metabolic process
*SUL1*	Ala^99^-Val	Sulfur assimilation
*HYR1*	Lys^172^-Glu	Cellular response to oxidative stress
*GTO2*	Trp^172^-Leu	Glutathione metabolic process
*SUL2*	Ala^809^-Ser	Sulfur assimilation
*ECM5*	Asn^504^-Lys	Cellular response to oxidative stress
*SAM3*	Glu^570^-Lys	S-adenosyl-L-methionine transport
*SSE1*	Asp^691^-Gly	ATPase component of the heat shock protein Hsp90 chaperone complex
*SSU1*	Met^19^-Val	Sulfite transport
*CYS4*	Ser^504^-Asn	Cysteine biosynthesis
*GSH1*	Ser^496^-Arg	Glutathione biosynthetic process
*STR2*	Gln^403^-Lys	Cystathionine biosynthesis

Certain classes of variants, such as InDels, are expected to have dramatic consequences on gene products and, therefore, constitute particularly interesting candidates for contributing to phenotypic variation (Additional file 12: Table S8). In all, 1690 and 1697 InDels were found in P5 and P24, respectively, compared with the reference strain (Additional file 11: Figure S4B). Of the total number of InDels, 823 were found in common between strains. When examining the distribution of the unique InDels for each strain along the chromosomes, some (chrXVII, X, XI, XV) showed an enrichment of variants (Additional file 11: Figure S4C). Only 12% of the unique InDels for each strain were within the coding sequence. Within ORFs, the InDels with lengths that were multiples of three were highly enriched when compared with the noncoding sequence, which is consistent with the strong purifying selection against frameshifts (Additional file 11: Figure S4D). Those InDels in the coding sequence with lengths that were not multiples of three were classified mainly as functionally uncharacterized. This demonstrates that this group of genes are, on average, under lower purifying selection pressure [27]. We also detected the larger copy number of genomic regions between both strains (CNV). Seventy-two CNV were detected across the genome comparison made between both strains (Additional file 12: Table S8). Most of these CNV were classified mainly as transposable elements and subtelomeric regions. These results are in line with previous observations [27], which found very limited CNV in nonsubtelomeric regions and extensive variation in subtelomeric regions.

## Discussion

Several works have shown the marked importance of temperature on the growth of wine yeasts [28–31] and the influence of this environmental factor on determining the natural distribution of wild species [3]. A direct effect of lowering temperature is to slow down the metabolic activity of yeast cells, which accounts for reduced growth and longer fermentation processes [32]. Thus, unraveling the molecular and physiological mechanisms that allow better adaptation and growth at low temperature is interesting. In this study we followed a global approach by comparing transcriptomic, proteomic and genomic changes in two commercial wine strains, which were selected as having clear differences in their growth and fermentation capacity at low temperature. The selection of these strains was based on the maximum growth rate in a synthetic grape must (SM) during miniaturized batch cultures at different temperatures. The fitness differences of the selected strains were corroborated by directly competing during fermentations at optimum and low temperature. These competition experiments highlighted the better competitiveness of P5 *vs.* P24 only at low temperature.

Although wine fermentations are operated in the batch mode, the proteomic and transcriptomic changes between both strains were determined in the steady-state of continuous cultures at the same dilution rate. In batch cultures, the specific growth rate (μ) is strongly affected by temperature. This means that it is impossible to dissect temperature effects on transcription and translation from specific growth rate effects [8]. Recently, Vázquez-Lima et al. [33] used chemostat cultures to mimic the different phases of a typical batch wine fermentation, and showed the potential of this experimental approach to systematically study the effect of environmental relevant factors such as temperature.

A global transcriptomic analysis has revealed key changes in the sulfur assimilation pathway at low temperature, with the up-regulation of key genes in both strains. This pathway incorporates extracellular sulfate into several key sulfur-containing compounds, including methionine, cysteine, homocysteine and S-adenosylmethionine (SAM) [34]. The biosynthetic genes of this pathway are controlled by a complex regulatory system, whose main transcriptional activator is Met4p. Met4p is recruited to specific promoters by site-specific DNA-binding transcription cofactors Met31p, Met32p and Cbf1p [35]. The activity of this pathway has a widespread influence on other cellular pathways, some of which have a huge potential impact on adaptation at low temperature, such as in the phospholipid (PL) biosynthesis pathway. Changes in the phospholipid composition of cellular membranes as a response to low temperature have been widely reported [28, 29, 36, 37]. Phosphatidylcholine (PC), the major phospholipid (at least 30% of total PLs), is synthesized *de novo* from another PL, phosphatidylethanolamine (PE), in three SAM-consuming methyltransferase reactions catalyzed by Opi3p and Cho2p [38]. The enzymatic genes of this pathway are repressed by Opi1p, a protein that directly senses the levels of phosphatidic acid (PA), a precursor of PL biosynthesis. Hickmann et al. [34] reported coordinated regulation of the sulfur and phospholipid pathways with Met4p activating the genes involved in producing SAM and Opi1p repressing some of these genes. This coordinated regulation between both transcription factors ensures that cells maintain the requirement of methylation during the biosynthesis of cell membrane phospholipids. Thus we hypothesize that the higher demand of PC at low temperature [37] increases the requirements of SAM for this biosynthesis, requiring the activation of the entire sulfur metabolism pathway. The better fitness of strain P5 at low temperature is consistent with the global transcriptional up-regulation of enzymatic genes and some transcription factors in comparison to strain P24. The greater transcriptional activation of this route in P5 also correlated with the presence of higher concentrations of some Met proteins, such as Met10p and Met17p. However, P24’s inability to synthesize SAM requirements at low temperature can be counterbalanced by the presence of this compound in the growth medium, which showed a similar growth rate to strain P5. These data also support the higher internal demand of SAM for growing at low temperature.

Greater activation of the sulfur assimilation pathway may also have a huge impact on other metabolic processes, such as the synthesis of the molecules involved in oxidative stress response. Thioredoxin and glutathione /glutaredoxin pathways are universal systems to maintain the redox homeostasis of the cell. The oxidized disulphide form of thioredoxin is reduced directly by NADPH and thioredoxin reductase, whereas glutaredoxin is reduced by glutathione (GSH) using electrons donated by NADPH (Figure 5). Thus according to our data, the coordinated up-regulation of the genes involved in the sulfur and glutathione pathways may lead to higher intracellular concentrations of glutathione, whose protective effect may contribute to improve the fermentation process. In a recent transcriptomic comparison of four wine strains showing different fermentation performances, Treu et al. [39] correlated the induction of the genes involved in the biosynthesis of sulfur amino acids with the strains showing better fermentation performance. Specifically, the higher expression of these genes, determined by the cooperation of TFs Met32p and Hap4p, contributed to more efficiently face stress induced by a high ethanol concentration and to improve strain fitness to starvation [40], which resulted in better fermentation performance. Not many reports have correlated low temperature and oxidative stress. Zhan et al. [41] reported increased transcript levels of antioxidant genes *SOD1*, *CTT1* and *GSH1* in a rapid downshift in the growth temperature of *S. cerevisiae* from 30°C to 10°C. Likewise, a previous proteomic study of our group [13] also detected an increase in Cys3p during wine yeast adaptation to low-temperature fermentation. Once again, the importance of glutathione biosynthesis in this cold adaptation is reinforced by growth data in the presence of both reduced (GSH) and oxidized glutathione (GSSG) in the culture medium. The GT of both strains significantly lowered, especially in strain P24, which showed similar values to P5. However, incubation in the presence of H_2_O_2_ clearly proved that strain P5 is more prone to cope with this stress oxidative. Once again, this is consistent with the up-regulation of practically all the enzymatic genes of both the sulfur and glutathione pathways, but also by the higher up-regulation of the key TFs of the route, such as Met32p and Met28p when compared with P24.

Once more, the proteomic comparison showed that strain P5 is better poised to deal with oxidative stress, as revealed by the higher concentration of peroxiredoxin Ahp1p and thioredoxin Tsa1p at low temperature. Likewise, the higher concentration of the Met proteins in strain P5 when compared to P24 also agreed with the transcriptomic data and reinforced the greater metabolic activity of the sulfur pathway in this strain. However, the transcriptomic and proteomic data did not always correlate directly. No MIPS category that was related to glycolysis and glucose fermentation was significant in the transcriptomic analysis, but the concentration of several proteins in this category changed at low temperature in both strains. Most of these proteins represented the enzymes of the lower part of the pathway (the trioses phosphate branch) and ethanol production. With a similar experimental set-up to this study, Quirós et al. [42] determined the distribution of metabolic fluxes during wine fermentations according to sugar concentration and temperature. In the upper part of glycolysis (the glucose 6-phosphate branch point), the C flux directed to glycolysis lowered at low temperature, which resulted in a higher C flux to the pentose phosphate pathway (PPP) and carbohydrate biosynthesis. These authors related this diversion of the C flux to these two minor branches with the higher biomass synthesis also observed at low temperature. Conversely in the lower part of glycolysis (the trioses phosphate node), the glycolytic flux was higher at low temperature, which resulted in a lower flux toward glycerol production. Biomass production is very high ATP-demanding. Thus, this higher biomass synthesis at low temperature may result in a shortage of intracellular ATP. The increase of glycolytic and fermentative enzymes leading to ATP generation may balance this drain. The simultaneous overexpression of these enzymes enhanced the glycolytic flux and fermentative capacity of *S. cerevisiae* [43]. In the comparison made of the strains, the extremely high concentration of Tdh1p (Glyceraldehyde-3-phosphate dehydrogenase) in strain P24 at low temperature was striking. Tdh1p catalyzed the reaction of glyceraldehyde-3-phosphate to 1,3 bis-phosphoglycerate, such as Tdh2p and Tdh3p, in the first step of the trioses phosphate branch. However Tdh2p and Tdh3p were detected in exponentially growing cells, whereas Tdh1p was detected primarily in the stationary phase [44]. It has therefore been suggested, but not confirmed, that Tdh1p may be involved in a process other than glycolysis because it is synthesized by cells in the stationary phase [25]. Likewise, the higher concentration of the proteins involved in translation machinery (Tef1p, Rpl31Ap) can also be correlated with a greater impairment of translation in P24 at low temperature. A recent study done by our group [45] showed that the better fitness of the cryophilic species *S. kudriavzevii* is given mainly by the enhanced translation efficiency of this species if compared to *S. cerevisiae*. This suggests that translational efficiency might be an important target of adaptation evolution when cells face changing environments.

Human intervention has subjected wine yeasts to multiple rounds of independent domestication and thousands of generations of artificial selection, which has driven to a phylogenetic lineage denominated by Liti et al. [26] as the Wine/European genetic clade. These authors also stated that despite a lineage formed by domesticated bred strains being expected to have lower phenotypic diversity, the Wine/European lineage showed similar or higher levels of diversity if compared to other clean lineages [46]. Our genomic data of the two wine strains confirm both concepts, phylogenetic proximity, but higher phenotypic diversity. They show a much smaller number of SNPs between them in comparison to reference lab strain S288c. However, despite a number of different SNPs representing less than 0.05% of the total genome, these genotype changes resulted in a clear divergent phenotype. Nonsynonymous changes in structural or regulatory genes can impact transcriptional regulation, mRNA stability or protein activity. A large number of mutations were detected in the genes of the sulfur and glutathione metabolic pathways. The changes in TFs such as *MET28* and *MET4,* which regulate the sulfur assimilation pathway, were particularly relevant. Hong et al. [47] analyzed the mutations produced in strains evolved with improved galactose utilization and concluded that the phenotypic changes observed in these evolved strains were the result of mutations in regulatory systems, which produced the overexpression and activation of some metabolic routes. Further work should be done in the future to evaluate whether these mutations cause the increase activity of this route.

## Conclusions

The combination of a detailed phenotypic analysis, e.g., involving transcriptome and proteome analysis, with genome sequencing is a powerful strategy to provide a clear link between genomic and phenotypic differences. We firstly selected two strains with different fitnesses at a low, but not at an optimum, temperature. Our data highlight the importance of the sulfur assimilation and glutathione pathways to explain the phenotypic differences between both strains. In order to distinguish which of the genetic differences seen in this study is responsible for difference in growth at cold temperatures, we are currently undertaking a new comparative genetic study based on the QTLs analysis of a hybrid population generated by crossing these two strains, following the approach described by Parts et al. [48].

## Methods

### Yeast strains

In this study, lab strain BY4743 and a collection of 27 *Saccharomyces cerevisiae* commercial wine strains were used. The industrial strains were kindly provided by Lallemand Inc. (France). These strains were typing by their interdelta sequences [49], and were thus named according to their delta pattern (from P1 to P27). Their corresponding commercial names are shown in Additional file 1: Table S1 and their enological features can be obtained from the company’s website (http://www.lallemandwine.com). Inocula were prepared by introducing one single colony from pure cultures of each strain into 5 ml of YPD medium (1% yeast extract, 2% peptone and 2% glucose). After 24 h of incubation at 30°C, the volume required to obtain a concentration of about 2 × 10^6^ cells/ml in the different media was used. The correct inoculation size was always confirmed by surface spread on YPD agar plates. These yeast suspensions were used to inoculate the different experiments as described below.

We also constructed a derivative P5 strain, which was labeled with the Green Fluorescence Protein (P5-GFP). The induction of this fluorescence protein allowed this reporter strain to be monitored by flow cytometry. One copy of the open reading frame (ORF) of gene *GAL1* was replaced with the deletion cassette GFP-*KanMX4* by the short flanking homology (SFH) method [50]. Plasmid pKT127 [51] was used as a template to obtain this deletion cassette. *S. cerevisiae* transformation was carried out by the lithium acetate method [52]. Transformants were selected by resistance to geneticin. Correct integration of the deletion cassette was confirmed by PCR using the primers upstream and downstream of the cloning site. Moreover, the fluorescence emission of the transformants was also tested after a 3-hour culture in YP-Gal medium (galactose 20 g/L, peptone 20 g/L, yeast extract 10 g/L).

### Media and growth conditions

The growth media selected for the experiments were SD (Yeast Nitrogen Base (YNB, Difco) supplemented with 20 g/l of glucose as the carbon source) and synthetic grape must (SM), which was derived from that described by Bely et al. [53]. The SM composition included 200 g L^-1^ of sugars (100 g L^-1^ glucose +100 g L^-1^ fructose), 6 g L-1 malic acid, 6 g L^-1^ citric acid, 1.7 g L^-1^ YNB without ammonium and amino acids, anaerobic factors (0.015 g L^-1^ergosterol, 0.005 g L^-1^ sodium oleate and 0.5 mL L^-1^ tween 80) and 0.060 g L^-1^ potassium disulfite. The assimilable nitrogen source used was 0.3 g N L^-1^ (0.12 g N L^-1^ as ammonium and 0.18 g N L^-1^ in an amino acid form). For the assays, the SD and SM media were inoculated as described above and were incubated at different temperatures (°C: 4, 8, 12, 15, 22, 28, 33, 37, 40, 42, and 45) in order to obtain the whole temperature range within which yeasts can grow.

To test the influence of some key metabolites of the sulfur assimilation and glutathione biosynthesis pathways on growth, SM was supplemented with one of these compounds: 0.2 mM of S-Adenosyl methionine (SAM, Sigma-Aldrich), 0.5 mM of Glutathione oxidized (GSSG, Sigma-Aldrich) and 1 mM of Glutathione (GSH, Sigma-Aldrich). To test differential stress oxidative resistance, yeast cells were incubated in PBS with 0.5, 1, 1.5, 2, 2.5, 3, 3.5 and 4 mM of peroxide of hydrogen for 1 h. After this stress oxidative shock, cells were centrifuged at 10000 rpm for 3 min at 4°C and inoculated in SD and SM as previously described.

Growth was monitored at 600 nm in a SPECTROstar Omega instrument (BMG Labtech, Offenburg, Germany). Measurements were taken every 30 min for 4 days after a 20-second pre-shaking for all the experiments. At low temperatures (4-15°C) however, microplates had to be incubated outside the spectrophotometer to be then placed inside before being measured (every 8 h for 14 days). Microplate wells were filled with the required volume of inoculum and 0.25 ml of SD or SM medium to always ensure an initial OD of approximately 0.2 (inoculum level of about 2 x 10^6^ cells/mL). Uninoculated wells for each experimental series were also included in the microplate to determine, and to therefore subtract, the noise signal. All the experiments were carried out in triplicate.

Growth parameters were calculated from each treatment by directly fitting OD measurements versus time to the reparameterized Gompertz equation proposed by Zwietering et al. [54]:


where y = ln(OD_t_/OD_0_), OD_0_ is the initial OD and OD_t_ is the OD at time t; D = ln(OD_t_/OD_0_) is the asymptotic maximum, μ_max_ is the maximum specific growth rate (h^-1^), and λ the lag phase period (h) [5]. GT was calculated using the equation GT = ln2/ μ_max_.

### Competition tests under microvinification conditions

Fermentations were performed at 28°C and 15°C with continuous orbital shaking at 100 rpm. Fermentations were done in laboratory-scale fermenters using 100 mL bottles filled with 60 mL of SM and fitted with closures that enabled carbon dioxide to escape and samples to be removed. Fermentations were monitored by the density of the media (g/L) using a densitometer (Densito 30PX, Mettler Toledo, Switzerland). Fermentations were considered complete when density reached 995 g/L. Yeast cell growth was determined by absorbance at 600 nm and by plating on YPD. The percentage of each strain competing throughout fermentation was monitored by both the replica plating from YPD to YPD-geneticin (G-418, Formedium) and by flow cytometry. The percentage of fluorescent cells was determined in a flow cytometer (Beckman Coulter Epics XL Flow Cytometer, Minnesota, USA) after GFP induction in YP-Gal (1% yeast extract, 2% peptone and 2% galactose) for 4 h at 25°C (no changes in population size were detected during this incubation). In all, 20000 cells of the sample were measured at a voltage of 700 V in FL1 FITC, which revealed the number and percentage of fluorescent cells and fluorescence intensity. The EXPO 32 ADC software was used for these measurements. The parameters measured with the cytometer were the number of fluorescent cells and average fluorescence intensity [55].

### Chemostat cultures and sampling

Continuous cultures were performed at 15°C and 28°C in an 0.5 L reactor (MiniBio, Applikon Biotechnology) with a working volume of 0.35 L. The dilution rate (D) of cultures was 0.028 h^-1^ at both temperatures. A temperature probe connected to a cryostat controlled temperature cultures. pH was measured online and kept constant at 3.3 by the automatic addition of 2 M NaOH. The stirrer was set at 100 rpm. The population inoculated in the chemostat was approximately OD = 0.2. Prior to starting the continuous culture, cells were allowed to grow at the same temperature as the continuous culture to achieve enough biomass in a batch phase. When the batch culture entered the stationary phase, the continuous culture was connected. Steady states were sampled only after all the continuous cultures had been running for at least five residence times and biomass values were constant. A volume of approximately 30 units of OD600 was centrifuged at 10000 g for 3 min at 4°C. After supernatant removal, cell suspension was washed with PBS, transferred to a 1.5-2 mL microcentrifuge tube and centrifuged again under the same conditions. The pellet was flash-frozen with liquid nitrogen and stored at -80°C until analyzed.

### Transcriptome analysis

RNA was isolated using the RNeasy Mini Kit (Quiagen) according to the manufacturer’s instructions. RNA was quantified spectrophotometrically with a NanoDrop ND-1000 (ThermoFisher Scientific) and integrity was determined by electrophoresis in 1% agarose gel. Next 2.5 μg of total RNA from each sample were linearly amplified and chemically modified with Amino-Allyl-UTP using the SuperScript RNA Amplification System (Invitrogen, Life Technologies). Then 5 μg of each amplified amino-allylRNA were indirectly labeled with Cy3 or Cy5 mono-reactive dyes (Amersham GE Healthcare™, Amersham UK) and were later purified with the RNeasy Mini Kit to remove nonincorporated dyes. Dye incorporation was monitored by NanoDrop ND1000. A mixture of 350–400 pmol of the two labeled samples was concentrated in a Concentrator Plus (Eppendorf™, Hamburg, Germany). Competitive hybridization was performed on a Yeast Array [56] (PCR-amplified ORFs of yeast S288c strain, Servei Genomica, Universitat Autonoma Barcelona, Spain) in AHC hybridization chambers (ArrayIt Corporation, CA, USA) at 42°C overnight (17 h). The prehybridization solution contained 3X SSC, 0.1% SDS and 0.1 mg/ml BSA; the hybridization solution contained 50% deionized formamide, 5X SSC, 0.1% SDS and 0.1 mg/ml of salmon DNA. Microarrays were washed manually with solutions containing decreasing concentrations of filter sterilized SSC 20× and SDS 10% (Sol.1: 1× SSC-0.2% SDS; Sol.2: 0.1× SSC-0.2% SDS; Sol.3: 0.1× SSC; Sol. 4: double deionized water). The signal intensities of Cy3 and Cy5 were acquired with a GenePix 4100A scanner (AXON, Molecular Devices, CA, USA) using the GenePix Pro v.6.1 software at a resolution of 10 μm.

The microarray data were derived from three independent experiments for RNA hybridization. The raw data with a global background subtraction were generated with GenePix pro 7.0. Analyses were done using the Acuity 4.0 software (Molecular Devices, CA, USA). The individual data sets were normalized to a log2 ratio value of 1. After normalization, data were filtered to remove spots flagged as not found. Only the spots with at least three replicates were considered. Finally, replicates were combined and their medians were calculated. Genes with 2-fold differences in the log2 ratio values were considered to have a significant differential expression if the p-values of the Student’s t-test were ≤0.05 after applying the Benjamini and Hochberg (BH) method to adjust for a false discovery rate (FDR) [57]. GO term Finder was used to group genes into functional categories, and is found in the MIPS Functional Catalog (http://mips.helmholtz-muenchen.de/funcatDB/).

### Proteomic analysis

#### Protein Extraction

The cell pellet was suspended in 150 μL of extraction buffer (25 mM TRIS buffer, pH 8, 8 M urea and protease inhibitor cocktail (1/200) (Thermo Scientific)) and was broken by vortexing (4 to 6 times, 30 s) in the presence of glass beads (Sigma-G8772) (an equivalent volume to that of the cell pellet). Glass beads and insoluble material were eliminated by centrifugation (10000 rpm, 10 min). To the supernatant, 150 μL of extraction buffer were added. Proteins were allowed to precipitate at -20°C for 1 h, and the precipitate was recovered after centrifugation at 10000 g for15 min. The pellet was washed with the 2-D Clean-Up kit (GE Healthcare). The final pellet was air-dried and solubilized in 25 μL of 7 M urea, 4% (w/v) 3-[(3-cholamidopropyl)dimethylammonio]propanesulfonate(CHAPS), 2 M Tiourea, 20 mM Tris and milliQ water. Insoluble material was removed by centrifugation (13000 rpm, 5 min). Protein concentration was determined by Bradford, with BSA used as a standard.

### Two-dimensional electrophoresis (2DE)

Soluble proteins were run in the first dimension using a commercial horizontal electrophoresis system (MultiphorII; Amersham Pharmacia Biotech). Then 100 μg of the protein sample were mixed with Destreak Rehydration Solution (GE Healthcare), dithiothreitol (DTT) (20 mM) and IPG buffer, pH 3–10 NL (GE Healthcare), and loaded onto Immobiline^TM^ DryStrip pH 3–10 NL, 24 cm (GE Healthcare). IPG strips were allowed to rehydrate overnight. Samples were run at 50 mA per strip. In the first step, voltage was ramped to 500 V during a 5-hour period, maintained at 500 V for another 5-hour period, re-ramped to 3500 V during a 9.5-hour period and was finally maintained at 3500 V for 5 h. After the first dimension, IPG strips were then equilibrated twice for 15 min in equilibration solution (0.05 M Tris–HCl, pH 8.8, 6 M urea, 30% v/v glycerol and 2% w/v SDS), first with 65 mM dithiothreitol (reduction step) and finally with 135 mM iodoacetamide (alkylation). The second dimension was done in a vertical electrophoresis system (Ettan DALTsix; Amersham Pharmacia Biotech) in a 12.5% (26 cm_20 cm_1 mm) polyacrylamide gel, where proteins were separated according to molecular size. The electrophoresis conditions were 1 W per gel until the dye front reached the bottom of the gel. Sets of three gels were used for each sampling time.

### Staining and image analyses

The staining protocol was performed as described by Blomberg et al. [58]. Gels were scanned using an Image Scanner UMAX, Amersham (300 dpi, 12-bit image), which allowed us to obtain spot intensities in pixel units. Images were analyzed using the PDQUEST software (Bio-Rad). Normalization was performed by the aforementioned software based on the total required in gel density to compensate the image differences caused by variations under the experimental conditions (e.g., protein loading or staining). Spot detection was implemented using the PDQUEST automated spot detection algorithm. The gel image showing the largest number of spots and the best protein pattern was chosen as a reference template of the image analysis, and the spots in the standard gel were then matched across all the gels. Matching software features were used to relate and compare sets of gels. Finally, in order to achieve maximum reliability and robustness of the results, a Student’s t-test was performed. This test allowed us to identify those sets of proteins that showed a statistically significant difference with the confidence level set at 95%.

### MS analysis and protein identification

Protein spots were excised manually and samples were digested with sequencing grade trypsin (Promega) [59]. The digestion mixture was dried in a vacuum centrifuge and re-suspended in 4 μL of 2% ACN, 0.1% TFA. A BSA plug was analyzed in the same way to control the digestion process. Next 1 μL of the digestion mixture was spotted onto the MALDI target plate. After droplets were air-dried at room temperature, 1 μL of matrix (5 mg/mL CHCA (Bruker) in 0.1% TFA-ACN/H_2_O (1:1, v/v)) was added and allowed to air dry at room temperature. The resulting mixtures were analyzed in a 5800 MALDI TOFTOF (ABSciex) in the positive reflectron mode (3000 shots per position). Five of the most intense precursors (according to the threshold criteria: minimum signal-to-noise: 10, minimum cluster area: 500, maximum precursor gap: 200 ppm, maximum fraction gap: 4) were selected for each position for the MSMS analysis. The MS/MS data were acquired using the default 1 kV MS/MS method. Previously, the plate and acquisition methods were calibrated with 0.5 μL of CM5. The MS and MS/MS information was sent to MASCOT via Protein Pilot (ABSciex). A database search was done on Expasy. Searches were done with tryptic specificity to allow one missed cleavage and tolerance on the mass measurement of 100 ppm in the MS mode and 0.8 Da for MS/MS ions. Carbamidomethylation of Cys was used as a fixed modification, while oxidation of Met and deamidation of Asn and Gln were employed as variable modifications.

Then 3 μl of each sample were diluted to 6 μl with 0.1% TFA, 2% ACN. Next 5 μl of each final solution were loaded onto a trap column (NanoLC Column, 3 μ C18-CL, 75 umx 15 cm; Eksigen) and desalted with 0.1% TFA at 2 μl/min for10 min. Peptides were loaded into an analytical column (LC Column, 3 μ C18-CL, 75 umx 12 cm, Nikkyo)equilibrated in 5% acetonitrile 0.1% FA. Peptide elution was carried out with a linear gradient of 5-40% B in 30 min (A: 0.1% FA; B: ACN, 0.1% FA) at a flow rate of 300 nl/min. Peptides were analyzed in a nanoESIqQTOF mass spectrometer (5600 TripleTOF, ABSCIEX). TripleTOF was operated in the information-dependent acquisition mode, in which a 0.25-s TOFMS scan from 350–1250 m/z was performed, followed by 0.05-s product ion scans from 100–1500 m/z on the 20 most intense 2–5 charged ions. The MS/MS information was sent to search the database with the PARAGON algorism using the ProteinPilot software, v. 4.5 (ABSciex).

### SOLiD sequencing

Genome sequencing of the selected strains was performed by 5500xl SOLiD sequencing. Genomic libraries were prepared following the manufacturer’s standard instructions. Emulsion PCRs were performed using the SOLiD™ EZ Bead™ Systems. Sequencing was carried out by 75 nt single read exact call chemistry (ECC) and following the manufacturer’s standard protocols. The LifeScope software (v2.5.1, Life Technologies) was used to map color space reads, including the Accuracy Enhancement Tool (SAET) along the EF.4 Ensembl reference *S.cerevisiae* S288c genome assembly. Then SNPs and small-sized InDels were identified using the LifeScope software. The DiBayes algorithm, with highest-stringency calling, was used for single-nucleotide variant calling. The SNPs and InDels specific of strains P5 and P24 were identified by comparing the list in the “vcf” format. CNV was identified by mapping the reads of P5 to P24. The average depth of read coverage was computed in nonoverlapping windows of size 69 bp, and was normalized by the genome-wide median coverage for each strain. The log2 values of these ratios were then calculated using CNVseq (http://tiger.dbs.nus.edu.sg/CNV-seq). Finally, only the CNVs with a length longer than 1000 bp were considered. The whole-genome sequences have been published in the Sequence Read Archive (SRA) database (http://www.ncbi.nlm.nih.gov/sra/) and are available with access number SRP048919.

### Statistical analysis

Data were analyzed with the Sigma Plot 12.5 software and the results are expressed as mean and standard deviation. To evaluate statistical significance, tailed t-student tests were applied with a p-value of <0.05. Benjamini and Hochberg (BH) correction was used for the transcriptomic and MIPS analyses. Phenotypic data were fitted to the reparameterized Gompertz model by nonlinear least-squares fitting using the Gauss-Newton algorithm as implemented in the nls function in the R statistical software, v.3.0.[60].

## Availability of supporting data

The data set supporting the results of this article is available in the Gene Expression Omnibus (GEO) Database repository GSE60140 (http://www.ncbi.nlm.nih.gov/geo/query/acc.cgi?acc=GSE60140) and in the Sequence Read Archive (SRA) database repository SRP048919 (http://www.ncbi.nlm.nih.gov/sra/?term=SRP048919). The data set supporting the results of this article is included in the article (and its additional files).

## Electronic supplementary material

Additional file 1: Table S1: Yeast strains used in this study. (XLSX 12 KB)

Additional file 2: Figure S1: Box plot representation of the μ_max_ distribution in each strain within the complete temperature range assayed. Growth was performed in SD (A) and synthetic must (B). Box legend: bar inside the box represents the median value, upper bar represents maximum of distribution, lower bar represents minimum of distribution, and the circle represents extreme data points. Dashed line denotes the median value of μ_max_ of the 27 strains within the whole temperature range assayed. (PDF 86 KB)

Additional file 3: Figure S2: Population dynamics of a mixed culture strains between P5 (green lines) and P24 (red lines) growing in minimal medium (SD). The percentage of each strain was determined by flow cytometry during fermentation (0, 24, 48, 72, 96,144 and 240 h) at 15°C (▲) and 28°C (●). (PDF 38 KB)

Additional file 4: Table S2: Significant up- and down-regulated GO terms in P5 during low temperature fermentation. Numbers of genes are provided in brackets. (XLSX 12 KB)

Additional file 5: Table S3: Significant up- and down-regulated GO terms in P24 during low temperature fermentation. Numbers of genes are provided in brackets. (XLSX 12 KB)

Additional file 6: Table S4: Significant up- and down-regulated GO terms in P5 during low temperature fermentation if compared with P24. Numbers of genes are provided in brackets. (XLSX 11 KB)

Additional file 7: Table S5: Significant up- and down-regulated GO terms in P5 during optimum temperature fermentation as compared with P24. Numbers of genes are provided in brackets. (XLSX 13 KB)

Additional file 8: Table S6: Differential gene expression analysis. (XLS 118 KB)

Additional file 9: Table S7: Number of proteins modified in 2-D gels in all the experiments. (XLSX 12 KB)

Additional file 10: Figure S3: Recovery after oxidative stress. Cells were subjected to oxidative stress with different concentrations (0–4 mM) of hydrogen peroxide for 1 h. The oxidative agent was removed and the growth curves of P5 (1) and P24 (2) were analyzed immediately in SD (A) and SM (B) at 28°C. (PDF 110 KB)

Additional file 11: Figure S4: Genomic analysis of strains. (A) Single nucleotide polymorphism (SNPs) population distribution. SNPs were classified according to genome localization and change in protein sequence (nonsynonymous variant). (B) Venn’s diagram of the InDels in both strains compared with the reference strain. The common InDels among strains are highlighted. (C) Distribution along the chromosomes of the unique InDels. (D) Distribution of the unique InDels present in the coding sequence according to their length. (PDF 20 KB)

Additional file 12: Table S8: Genomic comparison among strains. (XLSX 154 KB)
