# Supplementary figures and images for: Global phenotypic and genomic comparison of two Saccharomyces cerevisiae wine strains reveals a novel role of the sulfur assimilation pathway in adaptation at low temperature fermentations
Source: BMC Genomics. 2014 Dec 3;15(1):1059. doi: 10.1186/1471-2164-15-1059 (PMC4265444; doi:10.1186/1471-2164-15-1059)

**A**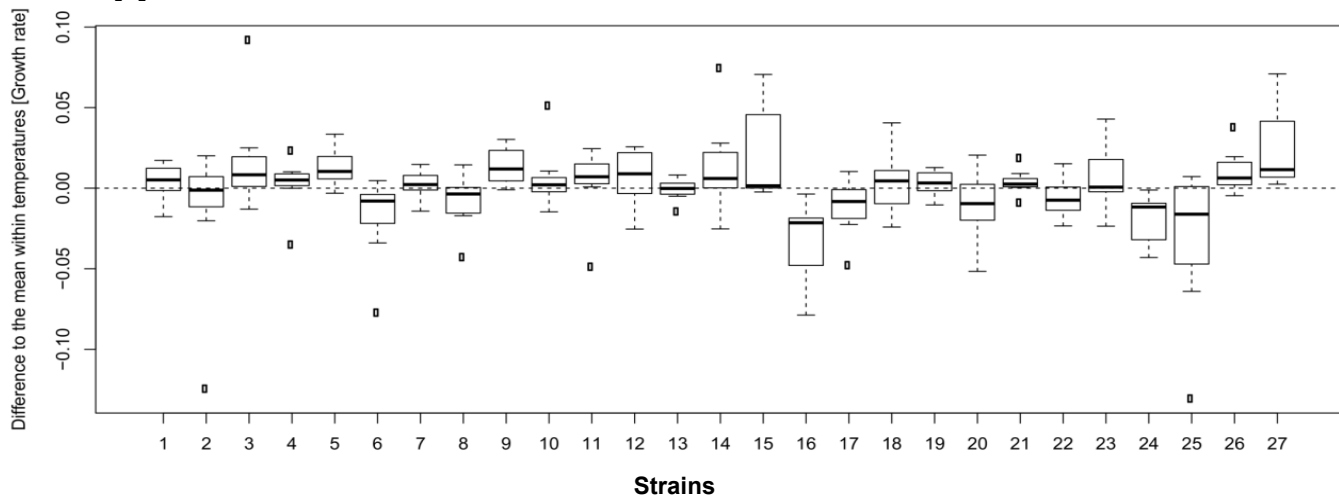**B**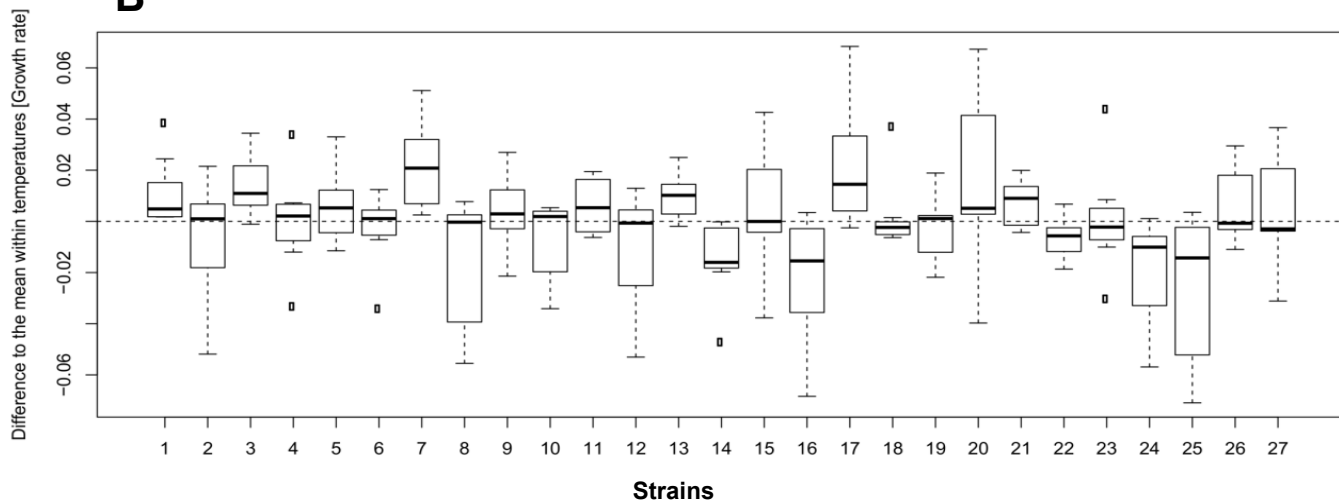

Supplement: Supplementary file 2 — Additional file 2: Figure S1: Box plot representation of the μmax distribution in each strain within the complete temperature range assayed. Growth was performed in SD (A) and synthetic must (B). Box legend: bar inside the box represents the median value, upper bar represents maximum of distribution, lower bar represents minimum of distribution, and the circle represents extreme data points. Dashed line denotes the median value of μmax of the 27 strains within the whole temperature range assayed. (PDF 86 KB) [file 12864_2014_6727_MOESM2_ESM.pdf]

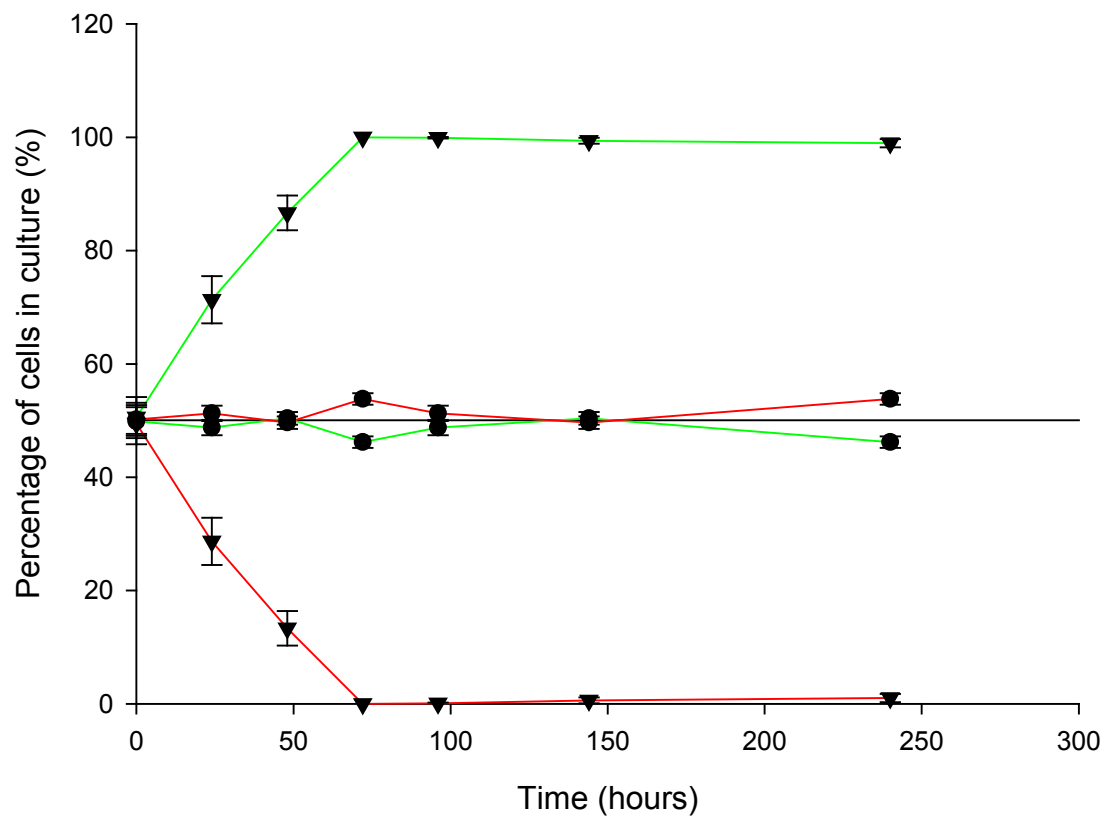

Supplement: Supplementary file 3 — Additional file 3: Figure S2: Population dynamics of a mixed culture strains between P5 (green lines) and P24 (red lines) growing in minimal medium (SD). The percentage of each strain was determined by flow cytometry during fermentation (0, 24, 48, 72, 96,144 and 240 h) at 15°C (▲) and 28°C (●). (PDF 38 KB) [file 12864_2014_6727_MOESM3_ESM.pdf]

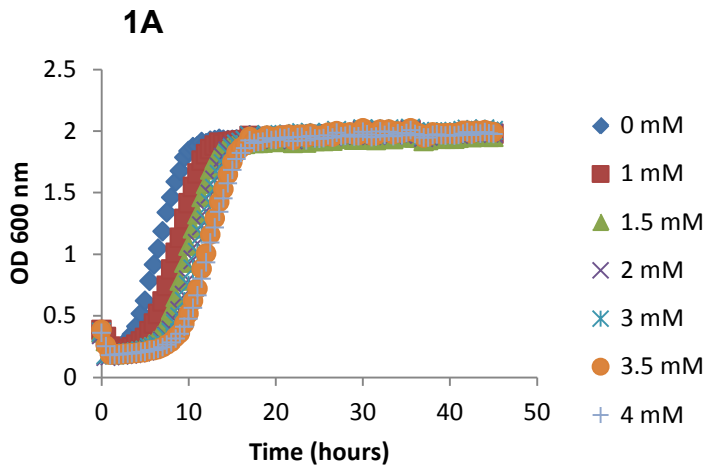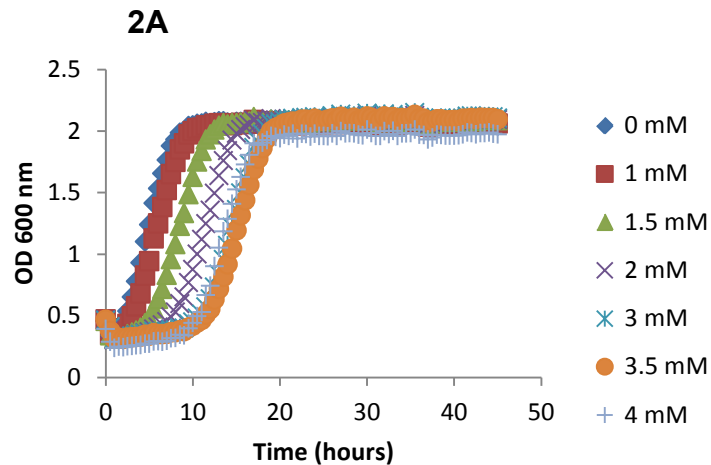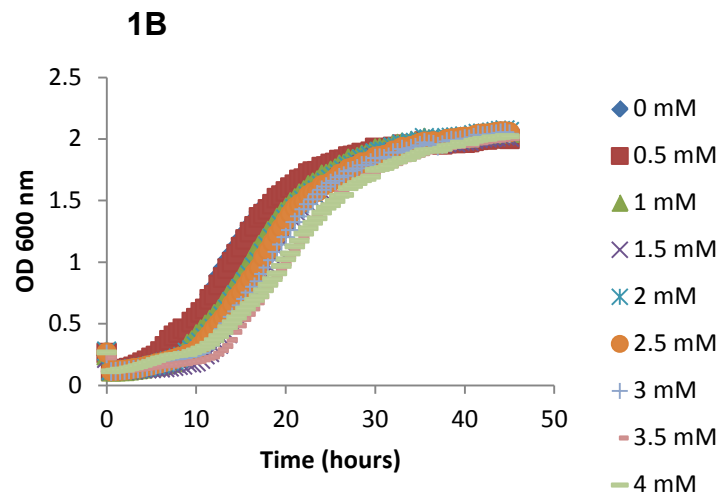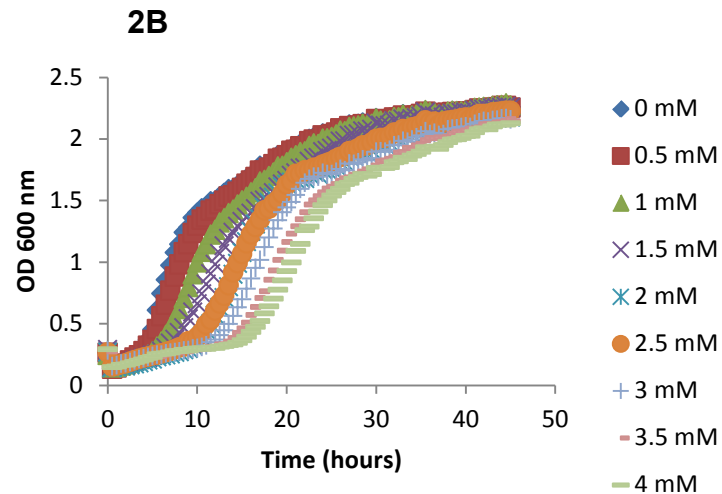

Supplement: Supplementary file 10 — Additional file 10: Figure S3: Recovery after oxidative stress. Cells were subjected to oxidative stress with different concentrations (0–4 mM) of hydrogen peroxide for 1 h. The oxidative agent was removed and the growth curves of P5 (1) and P24 (2) were analyzed immediately in SD (A) and SM (B) at 28°C. (PDF 110 KB) [file 12864_2014_6727_MOESM10_ESM.pdf]

**A**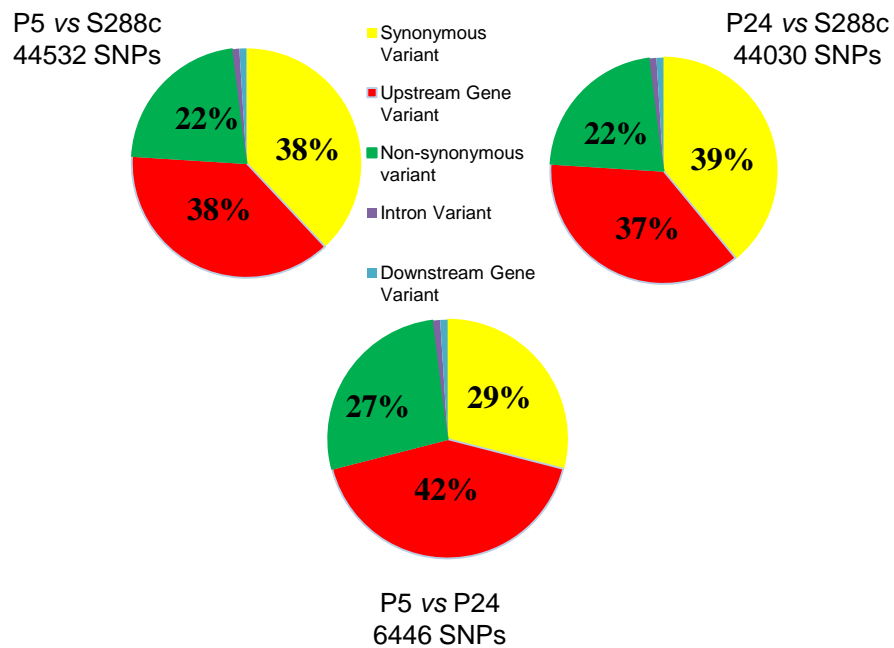**B**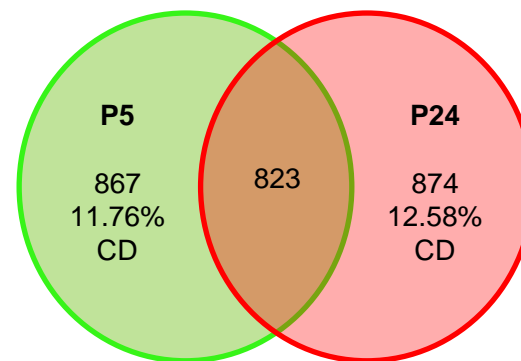**C**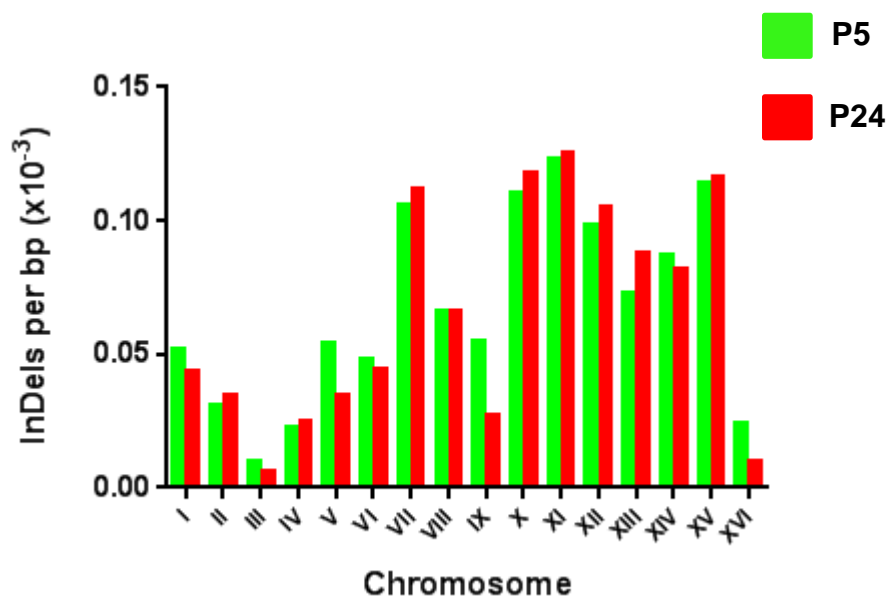**D**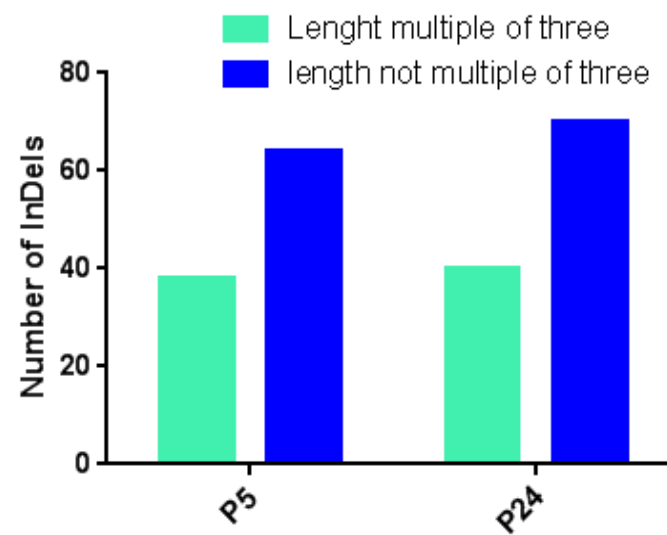

Supplement: Supplementary file 11 — Additional file 11: Figure S4: Genomic analysis of strains. (A) Single nucleotide polymorphism (SNPs) population distribution. SNPs were classified according to genome localization and change in protein sequence (nonsynonymous variant). (B) Venn’s diagram of the InDels in both strains compared with the reference strain. The common InDels among strains are highlighted. (C) Distribution along the chromosomes of the unique InDels. (D) Distribution of the unique InDels present in the coding sequence according to their length. (PDF 20 KB) [file 12864_2014_6727_MOESM11_ESM.pdf]
